# Relation of the work ability index to fitness for work in healthcare and public employees in a region of Northeastern Italy

**DOI:** 10.1007/s00420-023-02001-7

**Published:** 2023-08-16

**Authors:** Federico Ronchese, Francesca Ricci, Giulia Peccolo, Benedetta Persechino, Bruna Maria Rondinone, Giuliana Buresti, Corrado Negro, Massimo Bovenzi, Andrea Miani

**Affiliations:** 1 Occupational Medicine Unit, University Health Agency Giuliano-Isontina (ASUGI), 34148 Trieste, Italy; 2 Department of Occupational and Environmental Medicine Epidemiology and Hygiene, Italian Workers’ Compensation Authority (INAIL), Monte Porzio Catone, Via Fontana Candida 1, 00078 Rome, Italy

**Keywords:** Work ability index, Fitness for work, Job demands, Health status

## Abstract

**Purpose:**

Work ability indicates an individual’s capacity to match job demands according to his/her physical and mental conditions and work circumstances. Occupational physicians should take into consideration the global health status of a worker in order to correctly assess if he/she is fit for the job. The aim of this study was to verify the association between fitness for work evaluation and Work Ability Index scores, as well as individual factors (age, gender, and anthropometric characteristics) and work-related variables (job type, years of working duration).

**Methods:**

A cross-sectional study was conducted within the occupational health surveillance of health and public employers in the Friuli-Venezia Giulia region (2018–2022). The participants voluntarily agreed to answer the standard Work Ability Index questionnaire. Data were investigated by univariable as well as multivariable regression analysis.

**Results:**

The Work Ability Index of the workers included in the study (*N* = 6893) resulted negatively associated with age, female sex, and body mass index. It was averagely lower in nurses and assistive personnel, and the highest in medical doctors and public employers. The fitness for work assessments was also statistically related to WAI scores. The results obtained from the univariable and the multivariable analysis were consistent.

**Conclusions:**

The Work Ability Index is an efficient tool to measure an individual’s capability to sustain job demands, and can be taken into account to produce a correct fitness for work evaluation and consequently preserve workers’ health status.

## Background and objective

Working has always had a strong impact on health condition, affecting homeostatic regulation mechanisms, which are gradually weakened by age. However, there is evidence that age-related diseases can be more influenced by external factors, such as diet, job type, environment, and lifestyle, than genetic determinants (Friedman 2020). During the last century, impairment in the workplace has gradually become more frequently caused by cognitive and psychological disorders than somatic illness. This is due to the increase of professions requiring mental performance and reduction of weary jobs (Lebde et al. 2020).

Psycho-environmental stressors result in absenteeism, dissatisfaction, high turn-over, poor coping with colleagues, and can be measured with tools based on subjective perception of work and life quality and personal satisfaction, such as *Life Satisfaction Index*, *Work Ability Index*. Self-evaluation has resulted being a good predictor of future illness and work ability loss according to some authors (Denche-Zamorano et al. 2022).

“Work ability” can be defined as a worker’s well-being in the present and in the near future, and how capable will he or she be to perform his or her job, as regards to task requests and mental and physical resources. The Work Ability Index (WAI) developed by researchers of the Finnish Institute of Occupational Health is a commonly used tool to assess self-rated work ability, meant as a worker’s ability to manage tasks at a given moment, according to his/her physical and mental capacity (Tuomi et al. 1997). Individual factors may have an impact on the balance between personal resources and job demands, which may be perceived very differently according to the subject. Work ability is in fact also influenced by psychosocial factors, such as lifestyle, work organization, psychological conditions, insecurity and job dissatisfaction, work-related stress, as well as the self-perception of physical well-being (Gragnano et al. 2020). Negative predictors of work ability conservation are a poor work organization, high job demands, and high injury risk. Favorable factors are enhancement of professional skills, leaders’ and team support, health promotion, and physical activity (Lu et al. 2017).

Previously, studies have demonstrated the predictive ability of the WAI to identify workers at risk of long sickness absence, as well as early retirement, increased work disability and mortality (Palmlöf et al. 2019, Bethge et al. 2021). The aim of this study was to verify the association between individual factors (age, gender, anthropometric characteristics) and work-related variables (job type, job seniority) in a self-assessment such as the Work Ability Index, and to test its correlation with fitness for work evaluation.

## Methods

An Italian translation of the standard WAI questionnaire (Costa 2005; Costa and Sartori 2007) was used for the evaluation. It is composed of 7 key points to investigate the individual’s current work ability, work ability considering physical and mental job requests, the number of concomitant illnesses, number of days of sick leave during the last year, perceived work impairment due to health conditions, mental resources, and predicted work ability in the next years. The index is calculated summing up all single items. The total score ranges from 7 to 49 points, with higher scores corresponding to greater work capacity. In this way, work ability can be classified into poor (7–27), moderate (28–36), good (37–43), or excellent (44–49).

A cross-sectional study was conducted collecting data from January 2018 to July 2022. Workers were asked to participate in the survey on a voluntary basis. The WAI questionnaire was administered within the occupational health surveillance of health workers in the Friuli-Venezia Giulia region (FVG) in North-eastern Italy, that is, the University Health Agency Giuliano-Isontina (ASUGI), the University Health Agency of Central Friuli (ASUFC), the Health Agency of Western Friuli (ASFO), the Institute for Maternal and Child Health ‘IRCCS Burlo Garofolo’, and the National Cancer Institute of Aviano (IRCCS CRO). In the years 2021 and 2022, workers in Trieste’s town public administration were also included.

The data from the questionnaires was systematized using Microsoft Office Excel spreadsheet tools. Statistical analyses were performed using STATA v16.0 (Stata Corp LCC, Lakeway Drive, TX, USA). For measurable variables, arithmetic means, standard deviations, and frequencies were calculated. Differences between continuous variables were analyzed with Fisher’s F test, and categorical variables with a chi-square test. The WAI items were investigated with univariate and multivariate logistic regression analysis. A regression coefficient, standard error, confidence interval, and level of statistical significance were determined. A *p*-value < 0.05 was considered statistically significant.

## Results

The study population included 6893 workers (310 public service employers and 6583 healthcare workers), which were divided into 6 categories according to their job type. The first 5 groups included all healthcare workers, that is hospital administrative staff, sanitary technicians (e.g., radiology technicians, neurophysiopathology technicians, and physiotherapists), assistive personnel (healthcare personnel who assist patients in daily activities), medical doctors (including also pharmacists, veterinarians, biologist, and other higher degree professions), and nurses. The last category included all public service employees, as a homogeneous group. The study included fitness for work assessment, assessed through worker history, physical examination and clinical tests, of 1705 workers within the occupational health surveillance at the Occupational Medicine Unit of Trieste.

General and occupational characteristics of the study population are presented in Table 1.

**Table 1:** Characteristics of the overall study population

Factors	*N* (%)	
Gender		
Male	1751 (25.4)	
Female	5142 (74.6)	
Total		6893 (100)
Age (yr, quintiles)		
21–39	1452 (21.0)	
40–46	1545 (22.4)	
47–51	1306 (19.0)	
52–56	1344 (19.5)	
57–68	1246 (18.1)	
Total		6893 (100)
BMI (kg/m^2^, quintiles)		
15.4–20.8	1288 (20.0)	
20.9–22.7	1287 (20.0)	
22.8–24.7	1299 (20.2)	
24.8–27.7	1274 (19.8)	
27.8–63.8	1284 (20.0)	
Total		6432 (100)
Job categories		
Administrative staff	875 (12.7)	
Sanitary technicians	1288 (18.7)	
Assistive personnel	1193 (17.3)	
Medical doctors	706 (10.2)	
Nurses	2521 (36.6)	
Public service operators	310 (4.5)	
Total		6893 (100)
Work ability index		
Excellent	1925 (27.9)	
Good	3187 (46.2)	
Moderate	1520 (22.1)	
Poor	261 (3.8)	
Total		6893 (100)
Fitness for work assessment		
Fit	1196 (70.2)	
Temporarily partially fit	36 (2.1)	
Permanently partially fit	447 (26.2)	
Temporarily unfit	24 (1.4)	
Permanently unfit	2 (0.1)	
Total		1705 (100)

Data are given as numbers (%)

The correlation between individual factors, which correlate most strongly with work performance, (gender, age, BMI, years of working duration, and WAI) and the job category is presented in Table 2.

**Table 2:** Characteristics of the study population by job categories

	Hospital administrati ve staff (*n* = 875)	Sanitary technicians (*n* = 1288)	Assistive personnel (*n* = 1193)	Medical doctors (*n* = 706)	Nurses (*n* = 2521)	Public service operators (*n* = 310)	Total
Male* (n)	223 (12.7)	425 (24.3)	221 (12.6)	270 (15.4)	476 (27.2)	139 (7.9)	1754 (100)
Female* (n)	652 (12.7)	863 (16.8)	972 (18.9)	436 (8.5)	2045 (39.8)	171 (3.3)	5139 (100)
Age** (yr)	51.4 (8.8)	47.0 (10.0)	48.1 (8.3)	44.8 (10.6)	45.7 (9.3)	49.2 (10.4)	47.1 (9.6)
BMI**	25.0 (5.3)	24.7 (4.5)	25.4 (5.4)	23.8 (4.0)	24.4 (4.5)	25.4 (4.9)	24.6 (4.7)
(kg/m^2^)							
Years of working duration** (yr)	12.9 (10.5)	13.9 (10.4)	9.7 (8.4)	10.4 (8.9)	11.4 (8.9)	15.8 (11.6)	11.9 (9.6)
WAI** (units)	39.3 (6.2)	40.7 (5.3)	38.5 (6.3)	41.7 (5.5)	38.9 (5.9)	40.4 (3.9)	39.6 (5.9)

*Χ*^2^

*p < 0.001

*F* test between groups: **p < 0.001

Data are given as numbers (%) or means (SD)

In the study sample, 5142 were female (74.6%) and 1751 were male (25.4%). Sex was significantly correlated with the job (p < 0.001, Pearson’s chi-squared test). Although women represented the majority in all work categories, the highest proportions of female workers were among assistive personnel and nurses (over 81%).

The oldest workers were the administrative employees (51.4 ± 8.8), while the youngest were medical doctors (44.8 ± 10.6). In the univariate analysis, a statistically significant difference in age terms was found between and within different work categories (*p* < 0.001). With Bonferroni’s adjustment, the difference was confirmed between all groups (*p* < 0.05), with the exception of assistive personnel versus sanitary technicians and workers in public administration, and nurses versus medical doctors.

Anthropometric characteristics were also analyzed, and subjects’ body mass index (BMI) was categorized according to WHO criteria. The highest mean BMI were measured in assistive personnel (25.4 ± 5.4) and public service employees (25.4 ± 5.4), and both groups were found having a significantly higher BMI than medical doctors, nurses, and sanitary technicians (*p* < 0.001). The hospital administrative staff classified in third position (25.0 ± 5.3), but resulted significantly higher in comparison only to medical doctors (p < 0.05).

Another examined factor was the job seniority. The highest length of service was registered in public administration workers (15.8 ± 11.6), followed by sanitary technicians (13.9 ± 10.4), whereas the shortest in assistive personnel (9.7 ± 8.4). The difference was statistically significant (*p* < 0.001).

**Fig. 1:**
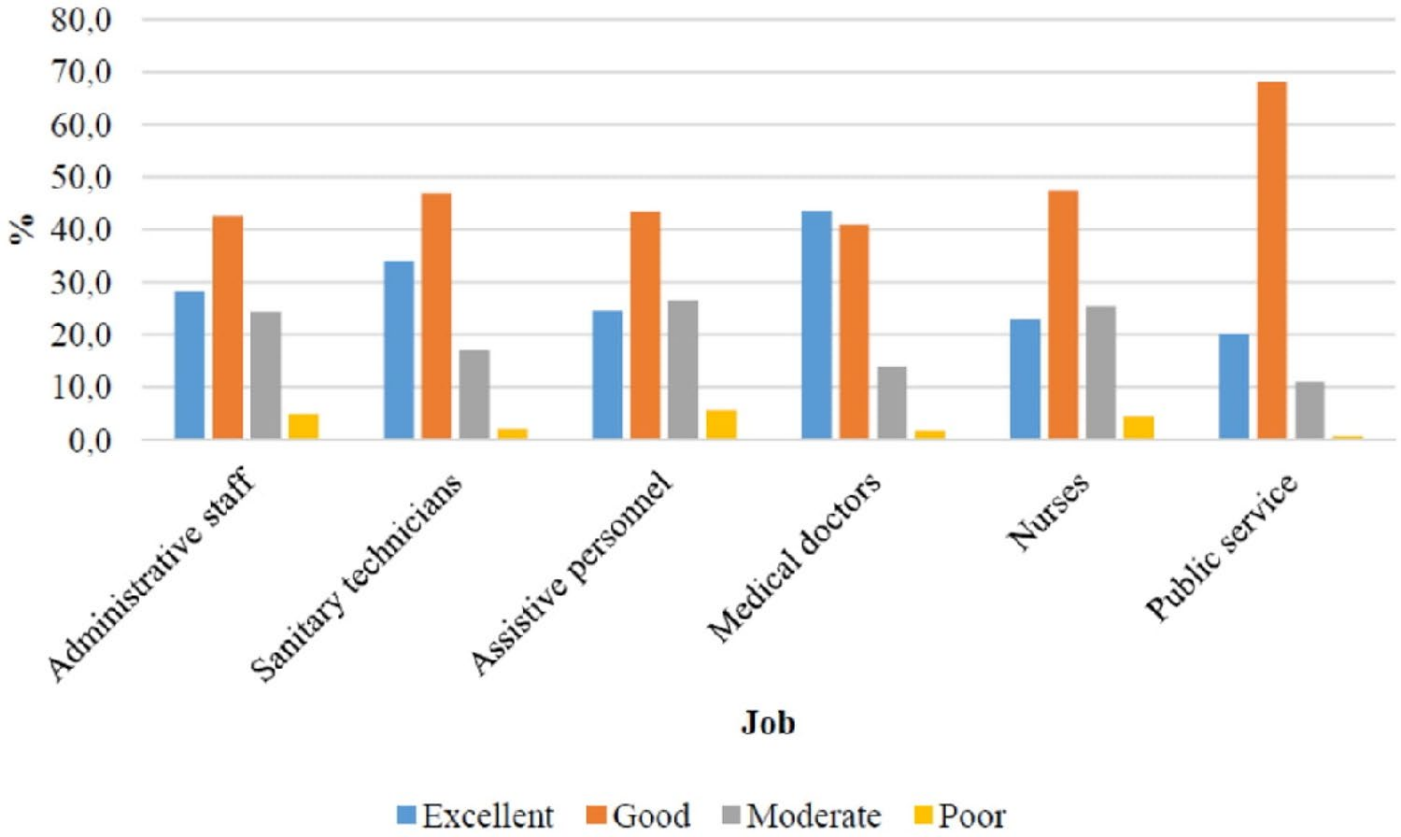
WAI results in work categories

The average WAI was ranked as “good” in all the different work categories. Its distribution is displayed also at Fig. 1. The scores resulted lowest among assistive personnel (38.5 ± 6.2) and nurses (38.9 ± 5.9), reaching statistical significance with the Bonferroni correction in comparison to the other work categories except when confronted with each other and with hospital administrative staff. On the other hand, the highest scores were found in medical doctors (41.7 ± 5.5), statistically significantly versus all other groups (*p* < 0.001).

Multiple regression analysis was used to identify significant predictors of work ability. The analysis was conducted testing separately the data corrected by job category and the data corrected by fitness for work assessment because the relationship between the two variables was too strong. In relation to WAI scores, where the variables showing significance (age, sex, and BMI) were used in the regression analysis, results indicated that 11.4% of work ability variance was explained by the independent variables (*R*^2^ = 0.114, adjusted *R*^2^ = 0.112), and the model was significant (*F* = 58.8, *p* < 0.001). The linear multiple regression analysis results in relation to WAI scores corrected by job category are shown in Table 3. Work ability gradually diminishes with age, by 1.9 points in the age range 40–46 to 4.3 points in the eldest workers compared to the younger ones (*p* < 0.001). Female workers have on average a lower WAI score than men (−1.8 points, *p* < 0.001). A progressive decrease can also be seen in the BMI distribution quintiles, although a significant correlation was found only in overweight ranges (BMI 24.8–27.7, *p* = 0.007; BMI > 27.8, *p* < 0.001).

**Table 3:** Linear multiple regression analysis results in relation to WAI scores corrected by job category

	Coefficient	Sd error	95% conf. interval	*p*-value
Cons	43.92	0.33	(43.28; 44.55)	< 0.001
Sex				
Male	–	–	–	–
Female	−1.76	0.17	(−2.09; −1.43)	< 0.001
Age				
21–39	–	–	–	–
40–46	−1.94	0.21	(−2.35; −1.53)	0.016
47–51	−2.58	0.22	(−3.02; −2.15)	< 0.001
52–56	−3.23	0.22	(−3.66; −2.79)	< 0.001
57–68	−4.31	0.23	(−4.76; −3.86)	< 0.001
BMI				
< 20.8	–	–	–	–
20.9–22.7	0.04	0.22	(−0.39; 0.47)	0.849
22.8–24.7	−0.08	0.22	(−0.51; 0.35)	0.706
24.8–27.7	−0.61	0.23	(−1.05; −0.17)	0.007
> 27.8	−1.43	0.22	(−1.87; −0.99)	< 0.001
Job				
Administrative staff	–	–	–	–
Sanitary technicians	0.67	0.25	(0.18; 1.17)	0.008
Assistive personnel	–0.81	0.27	(−1.33; −0.29)	0.002
Medical doctors	1.20	0.30	(0.62; 1.79)	< 0.001
Nurses	–1.04	0.23	(−1.49; −0.59)	< 0.001
Public service	0.42	0.39	(−0.34; 1.17)	0.282

Regarding job groups, it was confirmed that assistive personnel and nurses have on average a lower WAI score than other workers, respectively 38.5 ± 6.3 and 38.9 ± 5.9, while medical doctors result the fittest (41.7 ± 5.5), followed by sanitary technicians (40.7 ± 5.3). These results were statistically significant (*p* < 0.001).

**Fig. 2:**
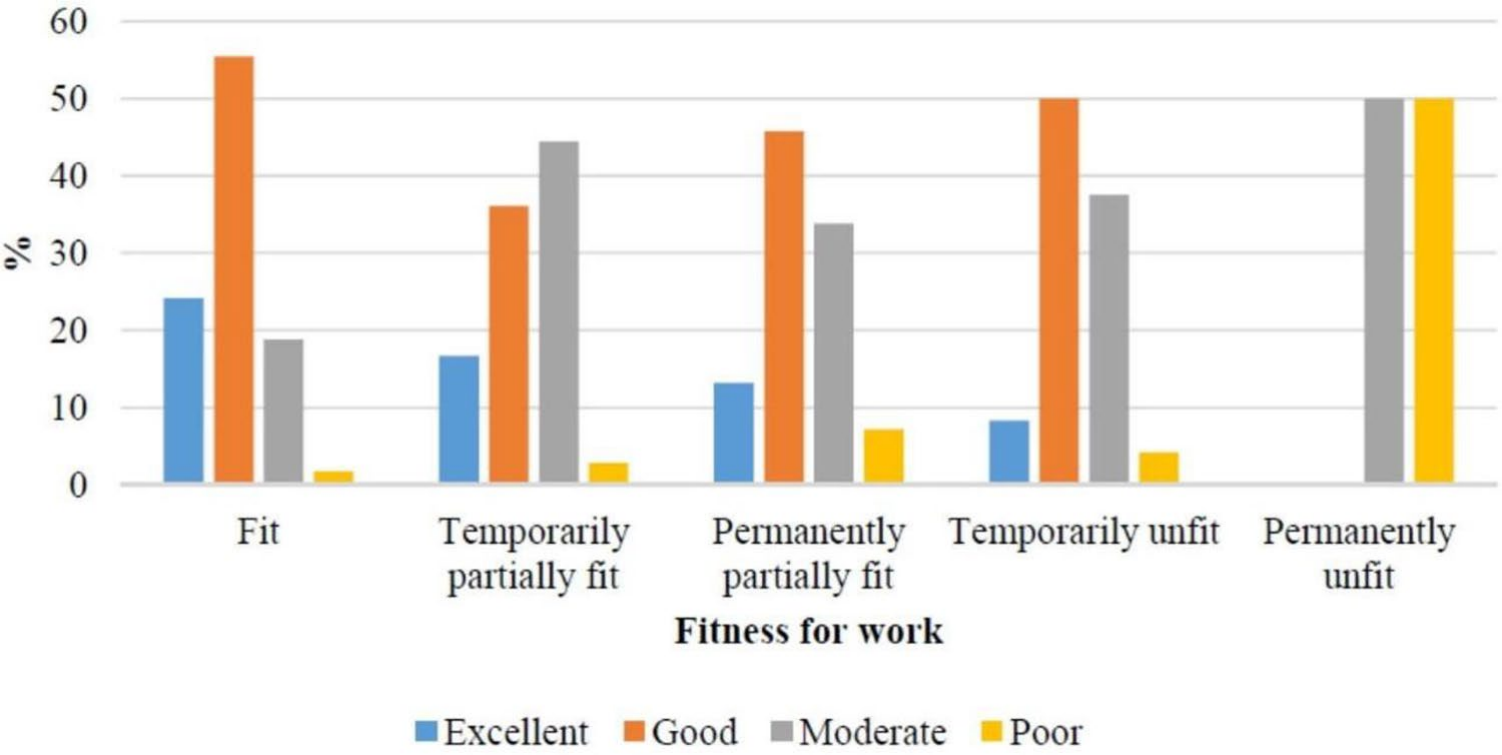
WAI and fitness for work

The regression model, which included the fitness for work assessment (N = 1705), but not the job category, showed that the factors significantly predicted work ability (*F* = 18.8, *p* < 0.001) and accounted for 12.8% of the variability of the WAI scores (*R*^2^ = 0.128, adjusted *R*^2^ = 0.121). A significant relationship was once again observed between work ability when accounting for demographic variables (age, sex, and BMI). A statistically significant correlation was found between fitness for work assessment and work ability. When compared with workers rated as fully fit, the WAI score decreases by 2.3 points in case of temporary partial fitness, by 2.4 points in case of permanent partial fitness, by 2.8 points in case of workers temporarily not fit for work, and by 9.1 points for individuals permanently unfit (*p* < 0.05). The linear multiple regression analysis results in relation to fitness for work assessment are shown in Table 4 and Fig. 2.

**Table 4:** Linear multiple regression analysis results in relation to fitness for work assessment

	Coefficient	Sd error	95% conf. interval	*p*-value
Cons	43.36	0.45	(42.48; 44.23)	< 0.001
Sex				
Male	–	–	–	–
Female	−1.86	0.29	(−2.42; −1.30)	< 0.001
Age				
21–39	–	–	–	–
40–46	−0.94	0.39	(−1.70; −0.18)	0.016
47–51	−2.35	0.40	(−3.13; −1.56)	< 0.001
52–56	−2.25	0.39	(−3.02; −1.47)	< 0.001
57–68	−2.92	0.41	(−3.73; −2.12)	< 0.001
BMI				
< 20.8	–	–	–	–
20.9–22.7	0.05	0.41	(−0.85; 0.75)	0.899
22.8–24.7	−0.44	0.41	(−1.24; 0.36)	0.283
24.8–27.7	−0.74	0.41	(−1.54; −0.06)	0.070
> 27.8	−1.19	0.40	(−1.98; −0.41)	< 0.001
Fitness for work				
Fit	–	–	–	–
Temporarily partially fit	−2.33	0.86	(−4.02; −0.65)	0.007
Permanently partially fit	−2.41	0.29	(−2.98: −1.86)	< 0.001
Temporarily unfit	−2.83	1.05	(−4.89; −0.78)	0.007
Permanently unfit	−9.07	3.58	(−16.10; −2.04)	0.011

## Discussion

The majority of the study population was female, which is representative of the gender distribution in the hospitals of the Friuli-Venezia Giulia, and similar to other studies on healthcare workers (Robbins and Davidhizar 2020).

Despite the growth of female prevalence throughout the last decades, professional disparities persist with women in the healthcare sector, with men dominating leadership roles (Senoo et al. 2020). The paradox between increasing representation without increasing professional success enhances the gender gap. The causes of this phenomenon can be sought in the different burdens of responsibilities at home, and therefore women are more likely to be influenced in career choice, salary, promotion, and leadership roles to ease family building, reinforcing gender stereotypes (Winkel Ford et al. 2021).

Women appear to be more affected by long-term musculoskeletal pain, which are among the most common occupational health problems (Howard et al. 2013), and studies have shown that workers taking sick leave due to this type of disorder may be associated to depression, social isolation and reduced income (Rashid et al. 2021). Women also suffer burnout at higher rates than men (Chesak et al. 2020). These widely recognized aspects explain well the averagely lower WAI scores reported for women in this study. The age distribution in this study does not differ from working populations in industrialized countries. World-wide the age pyramid is gradually being reversed, and this is mainly caused by increased life expectancy and decreased fertility and birth rates due to environmental, economic and circumstantial variables (Kontis et al. 2017). It is well known that aging is associated with a gradual decrease in biological functions, from a point of view both physical (e.g., visual acuity, muscular strength, and breathing resistance) and mental (e.g., memory functions, cognitive capacities, and psychomotor reactions). Mental rigidity and resistance to changes are also common in elderly workers, which may lead to relationship issues and discomfort on workplace (Dudel and Myrskylä 2017).

There are also positive aspects connected to aging, which may partially compensate for the negative ones. Elderly workers have on average a lower number of work injuries, which may however be more serious. Absenteeism is less frequent, but absences may last longer (ten Have et al. 2015). They also usually have more knowledge and experience, and are more reliable and capable of planning, and therefore they are able to maintain a good level of performance.

Consequently, it can be affirmed that the key issue is not the reduction of biological functions, but mainly the correct balance between job requests and functional ability and personal resources (Zacher et al. 2021). For these reasons, aging certainly affects work ability, as demonstrated in this study, but it is not the only or prevalent component.

In this study, overweight, and particularly obesity, was associated to lower WAI scores, meaning more difficulties in sustaining tasks at work compared with those of normal weight. Evaluating the fitness for work assessment, higher BMIs resulted in health-related work limitations, particularly regarding the ability to cope with the physical demands of work. Authors have hypothesized various possible mechanisms by which obesity could influence work ability, such as physical capacity being limited by the individual’s size, illness and comorbidity resulting from obesity, or discrimination and body image impairment, which may play a role in developing psychopathologies (Linaker et al. 2020).

Different job categories of healthcare workers are correlated to a variable risk of developing obesity, as it was shown in a study conducted by Kunyahamu et al. (2021). Similarly, in this study, it was found that professions such as assistive personnel and nurses are more likely to become overweight or obese. Features of these jobs, such as work shifts, night shifts, low educational attainment, low income, and disruptive working patterns, may directly or indirectly contribute to a greater risk of obesity (Kyle 2017).

Regarding the job seniority, the assistive personnel registered shorter length of service, which can be explained by the high turnover rates in this work category (Wakerman et al. 2019). On the other hand, workers employed in public service tend to maintain their job often throughout lifetime until retirement, hence on average more seniority. The other job category which tends to stay fixed in a workplace is the one of sanitary technicians because they are employed in few fields of work where their specific training is required, and therefore have lower possibilities of rotation.

The univariate analysis showed that WAI has a significant association with the job variable, in which physicians, followed by sanitary technicians, have a higher WAI score than other professional categories. On the other hand, nurses and assistive personnel report lower work ability. The multivariate analysis confirmed what emerged in the univariate analysis. The major loss of work ability in nurses and assistive personnel may be sought in the heavy physical activities their job usually requires, as well as psychological resources that tend to diminish over time. Furthermore, this decline affects negatively the motivation to work, which is recognized to be increasingly linked to mental and physical health status, stress management, and work organization (Rothmore and Gray 2019). By contrast, physicians are able to maintain a good work capacity, being less exposed to prolonged direct contact with patients (Arnsten and Shanafelt 2021). Other explanations may be hypothesized in a higher job satisfaction and a greater control over their duties, two determinants that are generally found more commonly among physicians than other healthcare professionals. This explanation would also be in line with previous studies showing a positive correlation between work ability and job satisfaction (Garzaro et al. 2022). By contrast, WAI scores appear substantially comparable for workers mainly involved in intellectual tasks, such as physicians, technicians, and white collars. Work satisfaction does not depend only on job type, but also on the expectations an individual has regarding it. This may explain why professional figures such as medical physicians, who underwent a long course of study to pursue a specific career, report higher psychological marks in reference to their job. The fact that job role in the healthcare sector influences the WAI score was seen to be supported to a similar extent to that seen in previously conducted studies (La Torre 2021). 

Surely one of the limitations of the study is represented by the heterogeneity of the sample. Most of the samples analyzed are made up of women (in agreement with the hospital working population) and the interviews come from different working sectors, so it appears necessary to evaluate the occupational risks of each occupation. For these reasons, it is difficult to reach a generalized conclusion.

Moreover, there are other variables that would be worth investigating, such as the night shift, but we did not have the exact number of shifts and the duration of them, so there was not possible to analyze it. It could be interesting to analyze also these variables in future studies.

## Conclusions

This study supports a relationship between work ability and fitness for work assessments among workers in the studied population. Both the individual and work-related features were significantly associated with WAI scores, in compliance with the theoretical principle of work ability, which is embodied by health based on functional ability and eventual diseases.

This study suggests that the WAI can be an effective tool to detect early physical and mental health impairments, and it can be helpful to preserve workers’ health status and provide an adequate fitness for work assessment. The results obtained from this study could be used to build a prospective study to monitor the variation of the WAI score and the fitness for work assessment in order to acquire further information for an appropriate management of human resources, and possibly for developing programs and services for extending work ability.

## Data Availability

The datasets generated and analyzed during the current sudy are available from the correponding author on reasonable request.
